# Knowledge and attitudes towards abortion from health care providers and abortion experts in Zimbabwe: a cross sectional study

**DOI:** 10.11604/pamj.2019.34.94.18107

**Published:** 2019-10-16

**Authors:** Mugove Gerald Madziyire, Ann Moore, Taylor Riley, Elizabeth Sully, Tsungai Chipato

**Affiliations:** 1 University of Zimbabwe, College of Health Science, Clinical Research Centre (UZCHS-CRC), Belgravia, Harare, Zimbabwe; 2 Guttmacher Institute, New York, USA

**Keywords:** Post abortion care, Induced abortion, Safe abortion, unsafe abortion, abortion law

## Abstract

**Introduction:**

Abortion in Zimbabwe is allowed to preserve the physical health of the woman, or in cases of rape, incest, or fetal impairment. Access even under these conditions is difficult and rare. We aimed to understand knowledge of the abortion law and attitudes towards abortion amongst health care providers’ and abortion experts in Zimbabwe as these can hinder access to safe legal abortion.

**Methods:**

In 2016, we conducted a Health Facility Survey (HFS) (n=227) among health care providers’ knowledgeable about abortion services in their facility in a census of facilities offering Post Abortion Care (PAC), and a Health Professionals Survey (HPS) among 118 abortion experts.

**Results:**

Twenty-five percent of providers and 47% of experts knew all four reasons under which abortion is legal in Zimbabwe. Amongst providers and experts, 31% and 50% respectively were misinformed about one or more legal criteria. Most providers and experts were in support of expanding the legal provision of abortion to cases when the woman’s mental health is at risk (65% and 79%, respectively) and if the woman is mentally incapacitated (66% amongst all). Seventy-one percent of experts recommend liberalizing the abortion law in order to reduce unsafe abortions.

**Conclusion:**

There is incomplete and sometimes inaccurate knowledge on the legal provisions for performing abortions in Zimbabwe amongst both health care providers and abortion experts. Incomplete knowledge of the law may be further reducing abortion access, highlighting the urgent need for educating health care providers on the legal status of abortion.

## Introduction

Zimbabwe has a restrictive abortion law, with legal abortion limited to preserve the physical health of the woman, rape, incest, or cases of fetal impairment [1]. In practice, however, access to legal abortion is difficult and rare [2]. Abortion providers and experts knowledgeable about abortion are gate keepers for women’s access to safe abortion. Therefore, it is helpful to understand their knowledge and perceptions of abortion in order to inform our understanding of the barriers to women’s access to safe, legal abortion in Zimbabwe. Abortion provider and experts’ perceptions about possible avenues for improving abortion access are also valuable as the country continues to grapple with addressing its high maternal mortality ratio of 651 maternal deaths per 100,000 live births [3], with complications from abortions accounting for 5.8% of maternal deaths, making it one of the top five causes [4]. Abortion also leads to substantial morbidity among women in Zimbabwe; of the approximately 65,300 abortions in Zimbabwe in 2016, of which 4 in 10 resulted in a complication requiring medical treatment [2] and 40% of women receiving post-abortion care (PAC) presented at health facilities with moderate to severe medical complications [5]. Unsafe abortions are therefore a critical public health issue in Zimbabwe, necessitating the need to better understand factors that may limit access to and use of the legal abortion in Zimbabwe. We aimed to understand providers’ and experts’ knowledge and misperceptions of the current abortion law, the extent to which they are in agreement with the current law, grounds under which they feel abortion should be provided and their recommendations for reducing unsafe abortion. Limited work has been conducted on attitudes of health care providers towards abortion in Zimbabwe. The earliest study done which explored knowledge and beliefs on abortion was published in 1999 and it looked at providers’ (nurses, doctors and social workers) knowledge of the law, their beliefs about when abortion should be allowed and factors influencing their attitudes [6]. Recent work by Chiweshe and Macleod using in-depth interviews with six health care providers in Harare found that providers framed women seeking abortions as transgressors of acceptable norms, irresponsible, manipulative and ignorant, based on cultural, religious, gender and trauma discourses that portray abortion negatively [7]. This paper improves on prior work by surveying health care providers in all facilities providing PAC nationwide and including experts on abortion.

## Methods

This work was conducted as part of a larger project to estimate national abortion incidence and assess the severity of post abortion complications among PAC patients in Zimbabwe [2, 5]. The methods used in these surveys were an adaptation of the Abortion Incidence Complications Methodology (AICM) applied in over 25 countries [8]. Knowledge and attitudes towards abortion were assessed using a Health Facility Survey (HFS) which was conducted with health care providers in charge of units offering PAC at all public, private and Non-Governmental Organization (NGO) health facilities that had the capacity to provide PAC. Out of 228 facilities with the capacity to provide PAC, 227 participated in the HFS. HFS respondents were selected based on their knowledge of the facility’s PAC services, which most often was a nurse/midwife (68%), senior nurse (27%), or doctor/hospital administrator (5%). To be eligible for the survey, the respondent had to have worked at the facility for a minimum of six months. Face-to-face structured questionnaires were administered by the Provincial Reproductive Health Officers who are trained health professionals working for the Ministry of Health and Child Care (MoHCC). Information was collected on PAC caseloads and services within the facility as well as the respondent’s knowledge of the abortion law, their opinions about the abortion law, and their opinions on how to reduce unsafe abortion in Zimbabwe. Following extensive review by Provincial Reproductive Health Officers, the HFS questionnaire was then pretested at two district hospitals, with interviews conducted among hospital coordinators, supervisors and nurses in the gynecological wards [9].

A Health Professionals Survey (HPS) was also conducted with experts knowledgeable on abortion to collect information on abortion-related complications and women’s access to treatment. A purposive sample of 125 experts covering all provinces was assembled by the study team which included representatives from the MoHCC. The list included doctors, nurses, midwives, policy makers, lawyers, pharmacists, traditional health workers, village chiefs, non-governmental organization (NGO) staff, and other individuals well-placed to know about women’s behaviors and outcomes in seeking abortion. The list also aimed to have sufficient representation of experts with knowledge of rural areas. Three-quarters of the sample were health professionals, and the remaining quarter were selected for their knowledge of abortion and women’s health issues at the community level. Only one selected respondent refused to participate in the survey. Six HPS respondents were excluded from the final dataset as they were assessed as having low knowledge on the topic by both the interviewer and themselves. The final HPS sample was 118 respondents. No respondent was included in both surveys. Information was collected on their knowledge of the abortion law, their opinions about the abortion law, and their opinions on how to reduce unsafe abortion in Zimbabwe was sought [9]. A pretest was conducted among two medical professionals, a doctor and a nurse, who were knowledgeable about abortion. Given the limited number of experts on abortion, further pretesting was not conducted so as to preserve our sample.

Data were collected between August and October 2016. The study was approved by the Medical Research Council of Zimbabwe, the Joint Research Ethics Committee for the University of Zimbabwe, College of Health Sciences and the Parirenyatwa Group of Hospitals and the Guttmacher Institute’s Institutional Review Board. For a full description of the methodology for both the HFS and HPS, see Sully *et al.* 2018 [2]. Our analysis draws on data from both surveys. Results from the two data sets are presented separately. As health care provider’s data come from a census of health facilities, and abortion experts were purposively selected, standard errors are not provided for any estimates.

### Ethics approval and consent to participate

We obtained ethical approval from the institutional ethics board of the Guttmacher Institute (20 May 2016), the Medical Research Council of Zimbabwe (28 April 2016, approval number MRCZ/A/2061) and from the Joint Research Ethics Committee for the University of Zimbabwe, College of Health Sciences and Parirenyatwa Group of Hospitals (4 April 2016, reference number JREC/379/15).

## Results

### Characteristics of respondents

Health care providers interviewed had been working in their profession an average of 15 years. Experts on abortion had on average 14 years of work experience. Seventy-six percent of health care providers and sixty percent of abortion experts were female. Ninety five percent of health care provider respondents and 36% of abortion experts were nurses (Table 1).

**Table 1:** Characteristics of respondents in the health facilities surveys and the health professionals survey, Zimbabwe 2016

	Health care providers (HFS) %	Experts (HPS) %
**Gender**		
Male	23.9	39.8
Female	76.1	60.2
**Age**		
20-24	0.4	0.9
25-29	4.4	10.3
30-34	18.9	17.9
35-39	18.5	14.5
40-44	14.5	14.5
45-49	15.0	17.1
50+	28.2	24.8
**Position at the facility**		
Doctor	2.2	29.7
Nurse/Midwife	95.1	36.4
Hospital Administrator	2.7	5.1
Pharmacist	-	4.2
NGO workers	-	11.0
Community workers and leaders	-	8.5
Law, government, media	-	5.1
**Years of experience**		
Average number of years	15	14
N	228	118

### Respondents’ knowledge of the abortion law

Among both samples of respondents, there was incomplete knowledge of the current abortion law in Zimbabwe. Experts had greater knowledge of the abortion law than health care providers, but there were important gaps in all respondents’ knowledge. There was greatest awareness among respondents of rape as a reason for legal abortion under Zimbabwean law: 83% of providers and 95% of experts knew this was a reason. Only 71% of health care providers and 79% of abortion experts were aware that abortion is allowed in cases of physical risk to the woman’s health. There were lower levels of knowledge about incest as a criterion for abortion, with only 46% of providers and 67% of experts knowing this is a legal criterion (Table 2). And the legal criteria for which there was the lowest level of awareness was if the fetus would be handicapped/have a fetal anomaly only 47% of providers and 71% of experts were aware of this legal condition. Amongst the experts, 19% and 14% incorrectly believed that abortion is legally allowed if the girl is under age 16 and if she is mentally incapacitated, respectively (Table 2). In sum, 25% of providers and 47% of experts knew all four reasons; 22% and 30% knew three reasons; 33% and 15% knew two reasons; 18% and 6% knew of one reason, and 3% in both groups did not know any reason (Table 3).

**Table 2:** Conditions under which key informants believe abortion to be legal, Zimbabwe 2016

	Health care providers		Experts	
	%	N	%	N
If pregnancy is from rape	83.2	191	94.9	112
If the woman's physical health is at risk	71.0	163	78.8	93
If pregnancy is from incest	46.2	106	66.9	79
If the fetus would be handicapped/fetal anomaly	47.2	109	71.2	84
If a girl is under age 16	4.8	11	18.6	22
Economic reasons (e.g. cannot care for the child)	0.0	0	1.7	2
If the girl or woman is unmarried	0.0	0	1.7	2
If the woman is mentally incapacitated	7.0	16	14.4	17
If the woman's mental health is at risk	4.4	10	3.4	4
If pregnancy is from contraceptive failure	0.0	0	0.8	1
If the girl or woman is still in primary or secondary school	0.0	0	0.8	1
If the woman doesn't want the pregnancy	1.7	4	0.8	1
If the woman is HIV positive	19.8	46	26.3	31
Under no circumstances	--	--	1.7	2
Don't know	2	5	--	--
N		230		118

Note: Multiple responses were allowed

**Table 3:** Knowledge of abortion law among health care providers and experts, Zimbabwe 2016

	Health care providers (HFS)		Experts (HPS)	
	%	N	%	N
Knowledge of Abortion Law				
Did not know any conditions	3%	7	3%	3
1 conditions	18%	40	6%	7
2 conditions	33%	74	15%	18
3 condition	22%	50	30%	35
4 conditions	25%	56	47%	55
Misinformed about one or more legal criteria	31%	71	50%	59

### Respondents’ attitudes towards the current law

Most providers and experts supported access to abortion under the grounds specified in the law, but support varied based on the condition. If the woman’s health was at risk (96% and 95%), rape (95% and 94%), and fetal anomaly (94% and 93%) were the conditions that garnered the most support among providers and experts. Health care providers expressed less support for incest as a reason for legal abortion (85%), while support remained high for this reason among experts (94%) (Table 4). There were also high levels of support in cases that are not allowed under the current law. The majority of health care providers and experts believed abortion should be legal if the woman’s mental health is at risk (79% of providers and 65% of experts) and in cases of mental incapacitation (66% of both providers and experts). Just under one-third of providers supported access to legal abortion for adolescent women under age sixteen (33%) as well as for adolescent and young women still in school (31%). Among experts, almost half (49%) supported access to legal abortion for adolescent women under the age of sixteen and 35% were supportive of abortion if the girl was in school (Table 4). One-fifth of providers and two-fifths of experts were in favor of abortion if the woman did not want the pregnancy and 14% of providers and 31% of experts were in favor of abortion in cases of contraceptive failure (Table 4).

**Table 4:** Under what conditions should abortion be allowed according to health care providers and experts, Zimbabwe 2016

	Health care providers		Experts	
	%	N	%	N
If the woman's physical health is at risk	96.4	215	94.8	109
If pregnancy is from rape	94.7	212	94.0	110
If pregnancy is from incest	85.3	191	94.0	109
If the fetus would be handicapped/fetal anomaly	94.3	211	93.8	106
If the woman's mental health is at risk	78.5	175	65.2	75
If the woman is mentally incapacitated	65.8	147	66.4	73
If a girl is under age 16	32.9	74	48.7	56
If the girl or woman is still in primary or secondary school	31.2	70	34.8	40
If the woman is HIV positive	23.8	53	35.0	41
Economic reasons (e.g. cannot care for the child)	7.1	16	23.9	27
If the girl or woman is unmarried	3.1	7	12.7	15
If pregnancy is from contraceptive failure	13.9	31	30.8	36
If the woman doesn't want the pregnancy	17.8	40	39.1	45
N		223		118

Note: Multiple responses were allowed

### Recommendations to reduce the occurrence of unsafe abortion in Zimbabwe

The experts surveyed were well-positioned to suggest solutions to the problem of unsafe abortion. The majority (71%) recommended liberalizing the legal status of abortion in order to reduce the consequences of unsafe abortion. Over half (53%) recommended publicizing the health risks of unsafe abortion and 50% recommended improving the coverage and quality of PAC services to reduce the consequences of unsafe abortion. Forty-two percent recommended improving information and access to contraception, while 35% identified improving access to safe abortion services (Table 5).

**Table 5:** Suggestions from experts on how to reduce unsafe abortion in Zimbabwe, 2016

	%	N
Liberalize the legal status of abortion	71.2	84
Publicize the health risk involved in unsafe abortion	52.5	62
Improve the coverage and quality of PAC services	50.0	59
Improve information and access to contraception	41.5	49
Improve access to safe abortion services	34.7	41
Provide free reproductive health services	31.4	37
Further restrict the conditions under which abortion is legal	5.1	6
Prosecute unsafe abortions	6.8	8
Encourage male involvement in abortion-related services	9.3	11
Discourage premarital sex	9.3	11
Discourage abortion (e.g. through religious teaching)	5.1	6
No opinion	1.7	2
Stop prosecution of women self-inducing abortion	2.5	3
Restrict sale of misoprostol	3.4	4
Expand and improve youth focused services	1.7	2
Involve traditional providers and leaders	2.5	3
Improve sex education in schools and in community	1.7	2
Improve social support for unintended pregnancies	2.5	3
N		118

Note: Multiple responses were allowed

## Discussion

This study highlights gaps in the knowledge of health care providers and abortion experts on the conditions for legal abortion as well as their perceptions of the abortion law and experts’ opinions on how to reduce unsafe abortion in Zimbabwe. Less than half of the health care providers knew that incest and fetal impairment were legal grounds for abortion. Incomplete knowledge of the law may be hampering abortion access even in cases where women would qualify for legal and safe abortion services. This has implications of misinformation of the abortion law among the public if they receive incomplete information about accessing safe abortion services from their health care providers. Experts were more likely to know the law than providers, which is perhaps not surprising given that they are a deliberate sample of individuals knowledgeable on abortion issues. However, even among those most knowledgeable about abortion in Zimbabwe, there were still a surprising number of experts who were not aware of all the legal conditions for abortion.

While most providers and experts did not know all of the legal provisions, most of them expressed support for the conditions currently allowed under the law. This study shows a change in perception over time in Zimbabwe with more providers supporting the legal conditions when compared to the study conducted from 1999 which showed lower proportions of health workers who believed that abortion should be allowed for woman’s physical health (84%), fetal anomaly (68%) and incest (75%) [6]. This relationship between the law and attitudes is most likely bi-directional: the conditions under which the law allows abortion are most likely seen as the most legitimate reasons for having an abortion and so as a consequence, they are the conditions that are most supported by providers and experts.

There are also high levels of support among health care providers and experts to expand the provision of eligibility for legal abortion beyond the current criteria. For example, the majority of respondents knew correctly that the law did not include if the woman’s mental health is at risk, however, the majority supported this as an additional criterion for access to legal abortion (79% of providers and 65% of experts). Seven out of ten experts suggest liberalizing the abortion law in order to reduce unsafe abortion. This is in line with the findings by Kasule *et al.* where 75% of doctors, 52% of nurses and 53% of hospital administrators supported liberalizing the law [6]. Half of the experts in our sample also supported a curative response by improving the coverage and quality of PAC services. However, this would not in fact address the number of unsafe abortions. A smaller proportion (42%) supported upstream factors such as increasing access to contraception, which could in turn reduce unintended pregnancies (of which a quarter end in abortion) [2].

The strengths of this study include that it was nationally representative and involved all provinces in Zimbabwe and all facilities offering PAC. This study updates the evidence base on the knowledge and attitudes of health care providers as well as experts on abortion in Zimbabwe, who are key actors and potential gatekeepers in accessing legal abortions. It also has several limitations. We cannot quantify the impact on women’s access to legal services based on provider misinformation but can only hypothesize that misinformation has led to reduced legal access. There may be other important gatekeepers to abortion outside of the health system who were not included in this study design, such as police officers. We also did not capture women’s knowledge and perceptions of abortion legality in Zimbabwe, which could highlight misinformation or confusion in the general public regarding abortion access.

Given these low levels of knowledge of all conditions for legal abortion among providers, it is possible that women seeking legal abortions at these facilities might be turned away and not receive a safe, legal abortion, even if they might meet a condition currently allowed under the law. It is crucial that the health care providers who are trained to provide these services know the conditions for legal abortion and can provide these services to the full extent under the current law. Educational campaigns focused on increasing knowledge of the abortion law and de-stigmatizing abortion should be targeted at professional societies of doctors, midwives and nurses as well as health educators who can incorporate these topics into undergraduate and graduate medical and nursing curriculums.

Adoption of flexible abortion laws is associated with a reduction in a country’s maternal mortality ratio [10]. There is overwhelming support among experts to liberalize the abortion law and expand the conditions under which abortion is legal in Zimbabwe. It is also clear that the majority of providers support liberalizing the law, with especially high support for additional conditions such as the mental health of the woman. An inclusion of mental health in the abortion law would be in line with the World Health Organization’s (WHO) definition of health, which includes psychological health [11] as well as the definition of health used by the African Charter on Human and Peoples’ Rights [12]. Including mental health as a condition for legal abortion can also be easily implemented within the health system. For example, in Kenya, a woman’s mental health assessment would be determined by a trained health professional who does not need to have a specialty in psychiatry to determine whether a woman meets the mental health indications for abortion [13, 14].

Health care providers have played large roles in other country’s process of liberalizing the abortion law as has happened in South Africa (Choice of Termination of Pregnancy Act 1996) [15] and Ireland (Repeal to the 8^th^ amendment 2018) [16] which was strongly advocated for by health care workers. Likewise, there could be potential opportunities for advocacy and support among Zimbabwean health care providers. Any expansion of the law would have to be coupled with broad educational campaigns to address existing gaps in knowledge, as well as any new conditions, among the general public and specifically for health care providers in training and in-service. Unsafe abortion can persist when abortion is liberalized but knowledge remains low as in the case of Zambia where abortion has been legal since 1972, yet many women still have unsafe abortions [17]. This study highlights gaps in understanding and practice. There is not a clear system in place for how women can access a legal abortion in Zimbabwe. Implementation of the law is subjectively and differentially managed, with women’s access largely being dictated by the individual who happens to have decision-making power over her [18]. Clarity is needed for providers, as well as women, about how legal abortions may be procured and provided. Future research is needed on the factors that affect providers’ and policymakers’ attitudes on abortion.

## Conclusion

There is incomplete knowledge among health care providers and experts in Zimbabwe on the legal provisions for abortion access, potentially contributing to the persistence of unsafe abortion in the country. Both providers and experts mainly support the current legal conditions and the majority of experts support liberalization of the abortion law, especially when the woman’s mental health is at risk or she is mentally incapacitated. Incomplete knowledge of abortion legality highlights the urgent need for health care provider education as a key step in reducing morbidity and mortality associated with unsafe abortion.

### What is known about this topic

- Stigma is a significant barrier to accessing legal abortion;
- Legalizing abortion does not result in all women accessing safe abortion services.

### What this study adds

- Access to safe abortion can be improved by improving health care providers’ and abortion experts’ knowledge of the abortion law;
- Health care providers and abortion experts believe abortion should be allowed if the pregnancy poses a risk to the woman’s mental health.

## Competing interests

The authors declare no competing interests.
